# Critical Role of Pathologists in the Accurate Subclassification of Non-Small Cell Lung Carcinoma (NSCLC) for Targeted Therapies: Evidence- Based Practice and the Role of IHC Markers

**DOI:** 10.4172/2476-2024.1000e105

**Published:** 2016-12-31

**Authors:** Qing Kay Li

**Affiliations:** Department of Pathology, The Johns Hopkins Medical Institutions, Baltimore, USA

## Editorial

Lung cancer is one of leading causes of cancer-related deaths worldwide. Recent genetic study has identified several driver gene mutations, which leads to the development of targeted therapies and improvement of our knowledge in lung cancer. In 2015, the WHO classification of lung cancer and the IASLC (International Association of Study of Lung Cancer) have updated their criteria and recommendation in lung cancer [1]. They emphasize the importance of accurate subclassification of lung cancers for targeted therapy. Clinically, the majority of lung cancer patients present with locally advanced disease or with distant metastasis at the time of diagnosis, and surgical resection of the tumor for morphological evaluation may not be the option. Thus, fine needle biopsy of the tumor become an important approach for diagnosis and staging of lung cancer as well as for molecular characterization of the tumor [2]. However, lung cancer is a heterogeneous group of neoplasms and accurate diagnosis on small biopsies can be challenging [1,2].

Recent systematic reviews and meta-analyses have shown that interobserver disagreement rates on the subclassification of non–small cell lung cancer (NSCLC) are approximately 10-20% in resected specimens and 20-30% in small biopsy specimen without IHC stains [3]. The morphological heterogeneity of the lung cancer is also correlated with certain molecular alterations [1]. Therefore, it is necessary to introduce newly updated guidelines of WHO and IASLC into our daily practice to improve the accuracy of subclassification of NSCLC for targeted therapy, particularly in small biopsy specimens.

The 2015 updated WHO classification of lung cancer and IASLC emphasizes the critical role of pathologists and IHC markers in the accurate subclassification of NSCLC, and also recommend to preserve tumor tissue for molecular characterization of NSCLC [1]. During the process of subclassification of lung cancer, multiple IHC markers may be used. The most commonly used IHC markers include TTF-1, Napsin A, CK7, P63 and P40 [4]. In small biopsy specimens, the use of multiple IHC markers may cause the exhaustion of the tumor tissue, compromising the molecular characterization of the tumor. Therefore, an alternative approach of using IHC markers is necessary to meet the clinical recommendations.

Based upon the WHO and IASLC guideline and clinical demand, we recently have combined commonly used individual markers TTF-1, P40, and Napsin A into a novel triple marker, and use it in the subclassification of NSCLC [5,6]. In cases, a morphological diagnosis of adenocarcinoma (ADC) or squamous cell carcinoma (SqCC) cannot be made based on morphology alone, the triple marker (containing 1 squamous marker and 2 ADC markers) can be very useful to provide valuable diagnostic information. Our results have demonstrated that the triple marker has showed a sensitivity and specificity of 86.0% and 100% in lung ADCs, and a sensitivity and specificity of 100% and 97.1% in lung SqCCs [5,6]. The triple marker demonstrates a similar sensitivity and specificity as individual IHC marker. Our practice also indicates that the utility of a combined IHC approach is cost-efficient way in subclassification of lung cancer in daily practice. Furthermore, the triple marker improves the turnaround time, and has the advantage of using minimal tumor tissue.

Taken together, the current WHO and IASLC criteria as well as our practical approach demonstrate that pathologists play a critical role in the accurate subclassification of NSCLC for targeted therapies and in the era of personalized medicine.
